# Searching for magnetic compass mechanism in pigeon retinal photoreceptors

**DOI:** 10.1371/journal.pone.0229142

**Published:** 2020-03-05

**Authors:** Alexander Yu. Rotov, Roman V. Cherbunin, Anna Anashina, Kirill V. Kavokin, Nikita Chernetsov, Michael L. Firsov, Luba A. Astakhova

**Affiliations:** Sechenov Institute of Evolutionary Physiology and Biochemistry, Russian Academy of Sciences, St. Petersburg, Russia; Syddansk Universitet, DENMARK

## Abstract

Migratory birds can detect the direction of the Earth’s magnetic field using the magnetic compass sense. However, the sensory basis of the magnetic compass still remains a puzzle. A large body of indirect evidence suggests that magnetic compass in birds is localized in the retina. To confirm this point, an evidence of visual signals modulation by magnetic field (MF) should be obtained. In a previous study we showed that MF inclination impacts the amplitude of *ex vivo* electroretinogram (ERG) recorded from isolated pigeon retina. Here we present the results of an analysis of putative MF effect on one component of ERG, the photoreceptor’s response, isolated from the total ERG by adding sodium aspartate and barium chloride to the perfusion solution. Photoresponses were recorded from isolated retinae of domestic pigeons *Columba livia*. The retinal samples were placed in MF that was modulated by three pairs of orthogonal Helmholtz coils. Light stimuli (blue and red) were applied under two inclinations of MF, 0° and 90°. In all the experiments, preparations from two parts of retina were used, red field (with dominant red-sensitive cones) and yellow field (with relatively uniform distribution of cone color types). In contrast to the whole retinal ERG, we did not observe any effect of MF inclination on either amplitude or kinetics of pharmacologically isolated photoreceptor responses to blue or red half-saturating flashes. A possible explanations of these results could be that magnetic compass sense is localized in retinal cells other than photoreceptors, or that photoreceptors do participate in magnetoreception, but require some processing of compass information in other retinal layers, so that only whole retina signal can reflect the response to changing MF.

## Introduction

Migratory birds use the Earth’s magnetic field (MF) for navigation and orientation during their long-distance journeys between breeding and wintering areas, and are known to possess both a magnetic compass [1] and a magnetic positioning system, a map [2]. The sensory basis of avian magnetic compass still remains a puzzle. A large body of behavioral data indicates that the avian magnetic compass has several important properties: it is based on the inclination angle of the magnetic field lines rather than on the polarity of the field [1], it is light-dependent, and, moreover, it depends on the light spectral composition. In experiments on magnetic compass orientation, birds of different species were able to orient under UV, blue and green light and disoriented under yellow and red light [3–8, for a review see 9]. Based on this fact, the primary magnetoreceptor in birds is supposed to be localized in the retina of the eye, and the prevalent hypothesis describing the work of such a magnetoreceptor is the radical pair model [10, 11; reviewed in 12]. According to this model, the primary receptor molecules that perceive the magnetic field are the cryptochromes. So far most promising candidate for being the magnetoreceptive protein is cryptochrome 4 (Cry4) because of seasonal but not circadian oscillations in its synthesis, and since its expression was shown in the outer segments of the double cones and long-wavelength single cones of avian retina [13]. It is assumed that cryptochromes are orderly oriented with respect to the surface of the retina, hypothetically due to binding to membrane proteins. These molecules absorb the short-wavelength photons, starting reversible chemical reactions, whose yield of (hypothetical) final products depend on the direction of the vector of the external magnetic field. However, at present, essentially nothing is known about the molecular mechanisms that might enable cryptochromes to transduce neuronal signals to the brain [for a review see 14].

For clarifying the mechanisms by which retinal cells can respond to the change in the MF, the most direct way is to make electrophysiological recording from the retina. Earlier we have tested a possible modulating effect of MF on the light response of pigeon retina. We found a small but statistically significant influence of the MF direction on the amplitude of half-saturated retinal responses to blue, but not red flashes [15]. ERG is a complex sum of electrical responses of several heterogeneous layers of cells. Since photoreceptors are supposed to be the most likely source of magnetoreception, we undertook a new series of experiments in which the possible influence of the magnetic field on the photoreceptor component of ERG was selectively analyzed. Thus we were going to see whether photoreceptor cells would make a major contribution to the effect observed in our previous study with recording from the whole pigeon retina.

## Materials and methods

### Experimental animals

The experiments were carried out on the retina of domestic pigeons *Columba livia* (n = 14). The birds were purchased from the breeder (“Pigeons of the Northern Capital” voluntary association, Russia) and kept for about one week in the lab at a 12:12 h light/dark cycle and with free access to water and food. Pigeons were housed in an outdoor aviary measuring 300 x 400 x 200cm. The cages fulfill the EU directive specifications for holding cages. In their housing caches, the pigeons could move about freely. Food and water were provided ad libitum. Floors of cages and aviaries were covered with wooden flakes and two perches were installed at different levels. Euthanasia was achieved by decapitation, in order to avoid effects of injected substances on retina. Decapitation conforms to institutional guidelines for animal welfare and the laws on the involvement of animals in experimental research issued by the government of Russian Federation and represents the most commonly used techniques to obtain tissue samples. The animals were treated in according to the protocol approved by Institutional Animal Care and Use Committee of Sechenov Institute of Evolutionary Physiology and Biochemistry, Russian Academy of Sciences (4-4/en dated April 23, 2018).

### Preparations, perfusion and solution

**Fig 1:**
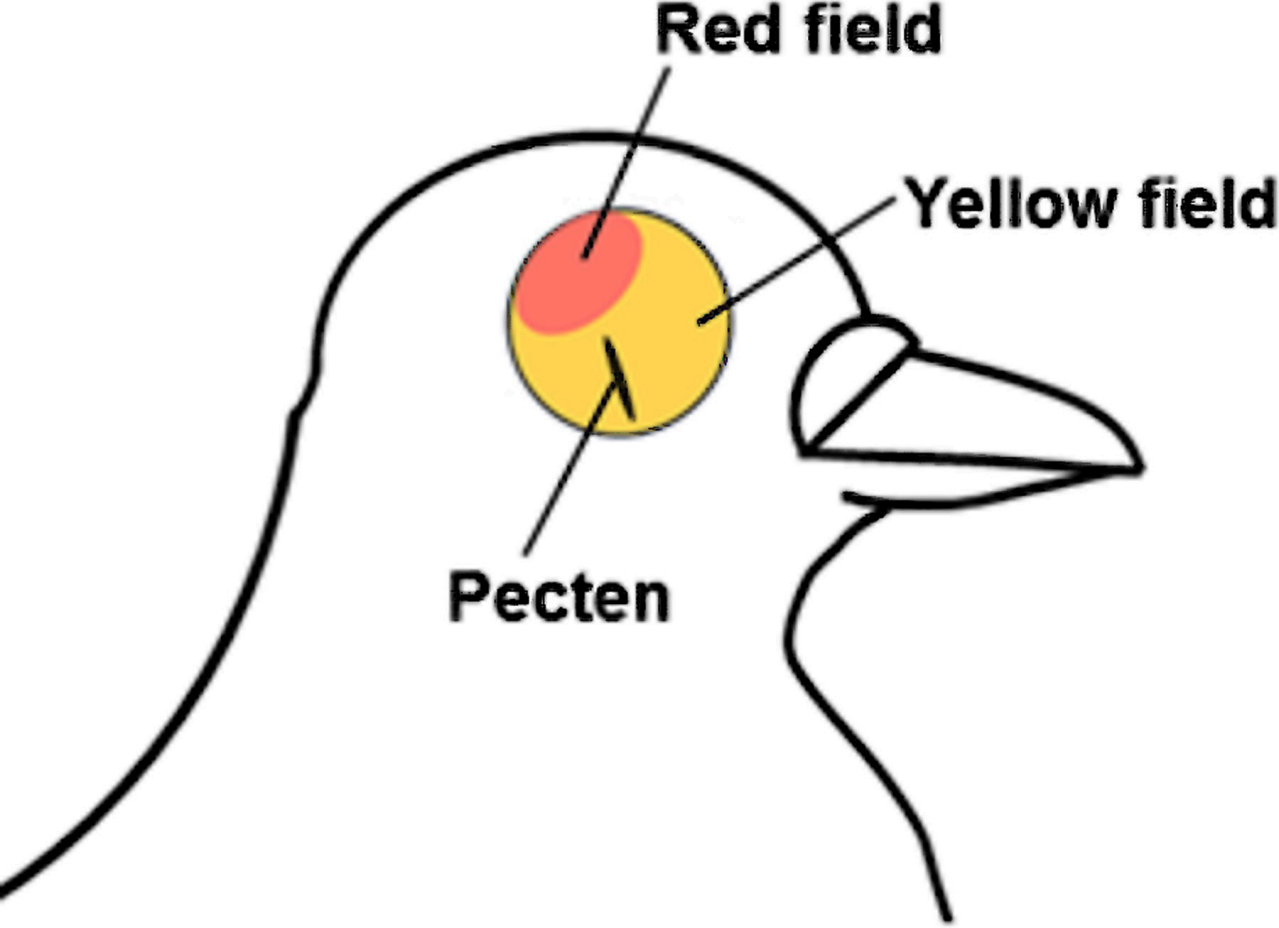
Scheme of fundus of the pigeon eye according to [16]. Positions of the red and yellow fields are shown.

Before the experiment, the birds were dark-adapted for about 1 hour. Their eyes were enucleated and the retinas were extracted under dim red light. Pigeon retina consists of two specialized areas (with different percentage of particular cone types): the “red field” in the dorso-temporal part of the retina and the “yellow field” in the remaining part (see Fig 1) [16]. For all tested eyes in this study retinae were divided into these two parts and recorded separately (see Result section).

Pigeon cone photoreceptors, like in all typical diurnal birds, contain colored and colorless oil droplets that act as cut-off filters reducing the sensitivity of cones in the short-wavelength part of the spectrum. The type of visual pigment in each particular cone can be inferred from the color of its oil-droplet [17]. In the pigeon’s retinal red field, the proportion of the total of red and orange oil-droplets, corresponding to long-wavelength sensitive pigment, is about 80% of the total cone count, which makes it look reddish. In the yellow field, red and orange oil-droplets make only 25% of the total cone count, making it yellow-looking. Therefore, the red and the yellow fields are characterized by different ratios of cone pigment types [18].

Tissue preparations and perfusion were made using Ringer’s solution for birds [19], containing in mM: NaCl 100, KCl 6, MgCl_2_2, CaCl_2_1, NaHCO_3_30, NaH_2_PO_4_1, Glucose 50; pH adjusted to 7.5. All chemicals were purchased from Sigma-Aldrich (St. Louis, USA).

The perfusion solution was bubbled with 95% O_2_/5% CO_2_ gas mixture and heated up to 37°C before passing through the perfusion chamber. Perfusion flow rate was 3 ml/min. Isolation of the photoreceptor potential from the whole retinal response was achieved by pharmacological means, commonly used in vision research: to cut-off the signal transduction from photoreceptors to second-order neurons, the perfusion solution was supplemented with 5 mM L-Aspartate. In order to suppress the glial (Müller cell) component of ERG, 0.1 mM BaCl_2_ was also added to perfusion solution [20–22].

### Electrical recordings and light stimulation

Transretinal voltage was recorded by *ex vivo* electroretinography [23]. Responses to the light were recorded across the isolated retina placed in an Ussing-type perfusion chamber, photoreceptor side down. Signals were captured by a differential amplifier (DAM50 Extracellular Amplifier, World Precision Instruments, FL, USA). Responses were recorded in the low-pass filtering at 1000 Hz at 10-ms digitization intervals.

The light stimulation system was based on a high-output light-emitting diode (LED). The stimulus intensity was controlled by switchable neutral density filters and LED current. Retinas were stimulated with 10-ms flashes of blue or red LED (λ_max_ = 470 or 630 nm, respectively). Data acquisition, stimulus intensity, and timing were controlled by National Instruments hardware and LabView software (National Instruments, Austin, TX).

### Magnetic field modulation

The MF around the preparation of the retina was controlled with coils creating a uniform magnetic field and measured with a magnetometer. A homogeneous magnetic field was created using three Helmholtz orthogonal coils with a diameter of about 30 cm. One pair of rings was used only to compensate for the earth's magnetic field along one of the horizontal directions. Coils were connected to DC laboratory power supply TK Lambda ZUP36-12 (TDK-Lambda Americas Inc. USA). Two other pairs of rings, oriented in the vertical and horizontal directions, created a uniform magnetic field in any direction in the vertical plane. To do this, the currents through the coils were controlled by a computer using a custom-made two-channel programmable current source. Thus, it was possible, in particular, to establish the vertical or horizontal direction of the magnetic field of 50 μT amplitude, which was correspondingly perpendicular or parallel to the plane of the retina. The switching time of the field direction was <1 ms. The magnetic field was independently controlled using a three-coordinate magnetometer based on the eCompass module LS303DHL from STMicroelectronics, connected to a computer through the STM32-Nucleo board. The measurement accuracy of the magnetic field was 1 μT.

To avoid any signal disturbances from ferromagnetic parts of experimental setup, the recording chamber with the retina preparation and the Helmholtz coils were placed at a distance from any iron-containing objects. A tight aluminum box protected the preparation from the ambient light and from the external electromagnetic interference. Stimulation system projected the flashes from LEDs onto retinal preparations via an optical fiber.

To further reduce electromagnetic noise near the preparation, the aluminum box was connected to the ground of the outlet. Typical noise spectra, recorded in the experimental room and inside the experimental chamber while all systems of the experimental setup are running are shown in S1 Fig (Supporting materials). Integration of the noise density in a range 0.1–10 MHz gives the total amplitude of the altering magnetic field near the preparation B = 8 nT, which roughly corresponds to conditions in which birds show magnetic orientation in behavioral experiments [24].

### Experimental protocol

**Fig 2:**
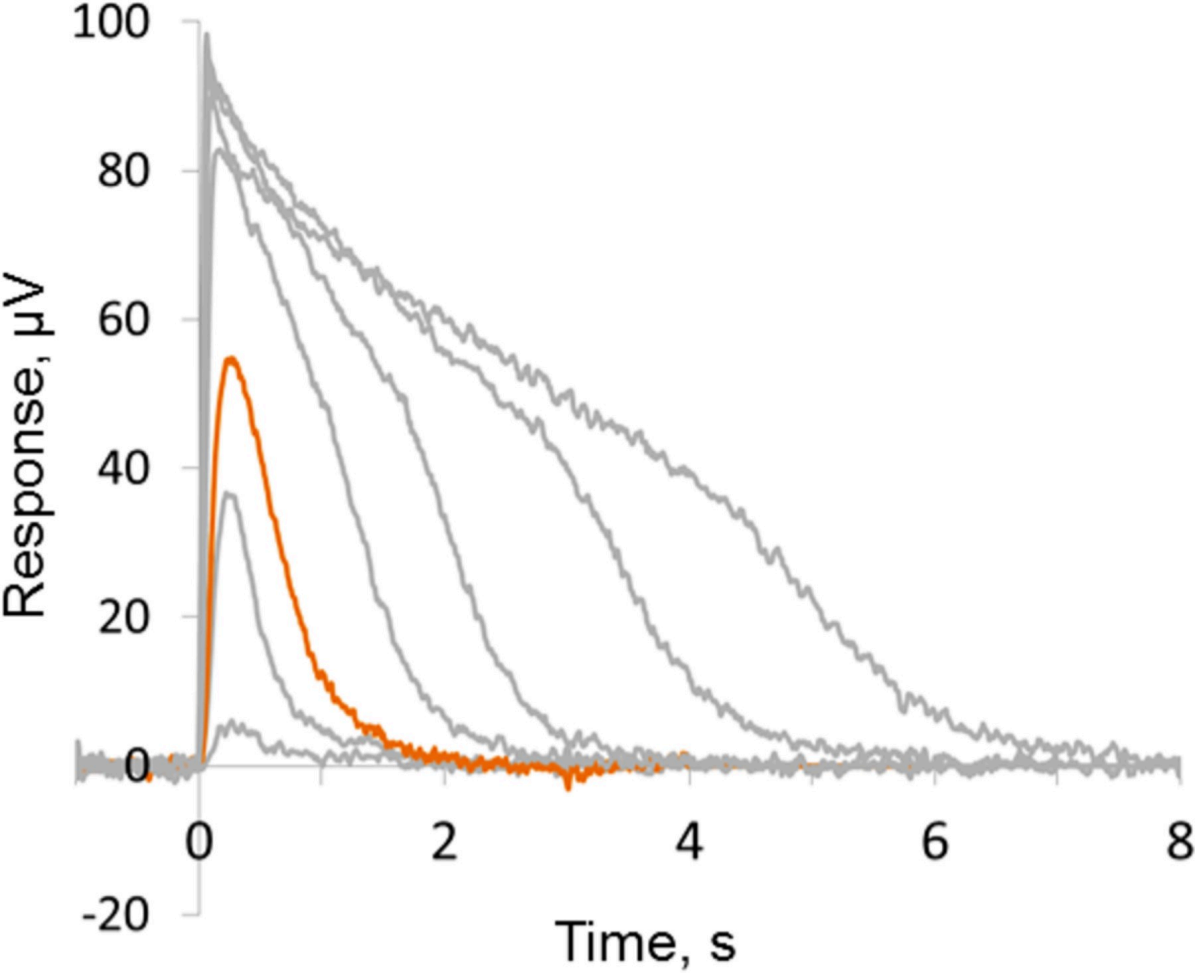
Typical set of photoreceptor responses of isolated pigeon retina to blue flashes of increasing intensities. Flash duration is 10 ms. The intensities of flashes were 3.7×10^6^, 4.67×10^7^, 1.17×10^8^, 4.17×10^8^, 1.32×10^9^, 4.36×10^9^ photons/mm^2^. Red curve indicates approx. half-saturated response (flash 1.17×10^8^) that was selected for further testing with modulated MF inclination.

In present study we investigated pharmacologically isolated full-field photoreceptor responses of the retina. Typical set of photoreceptor responses to blue flashes of increasing intensities is presented in Fig 2.

To detect a modulating effect of the MF on the photoreceptor responses, we used experimental protocol similar to the one described before [14].Two types of light stimuli were selected, blue (470 nm) and red (630 nm) because, according to behavioral data, birds are able to orient under blue, but not under red light [5–7].Therefore, one could expect the effect of MF modulation on responses to blue, but not to red stimulus. Light flashes were presented at two directions of the MF, orthogonal (inclination 90°) and parallel (inclination 0°) to the plane of the retinal preparations loaded into a perfusion chamber. The obtained photoresponses’ amplitude and kinetics were then compared. It should be emphasized that in the present protocol the magnetic field did not change during the signal recording. Therefore, there was no electromagnetic induction in the signal detection pathway caused by the electromagnetic system.

Before the start of the MF inclination modulation, the retinal preparations were initially stimulated by short blue and red flashes of increasing intensity (Fig 2) to determine the range of its sensitivity to light and choose the approximately half-saturating intensity of the flash. After that, we recorded four sets of responses (6 to 8 each): the first and third sets were recorded under 90°inclination of MF, the second and fourth ones, under 0° inclination. Time intervals between sets’ recordings varied from 46 to 371 s. Responses in every set were then averaged to increase the signal-to-noise ratio.

**Fig 3:**
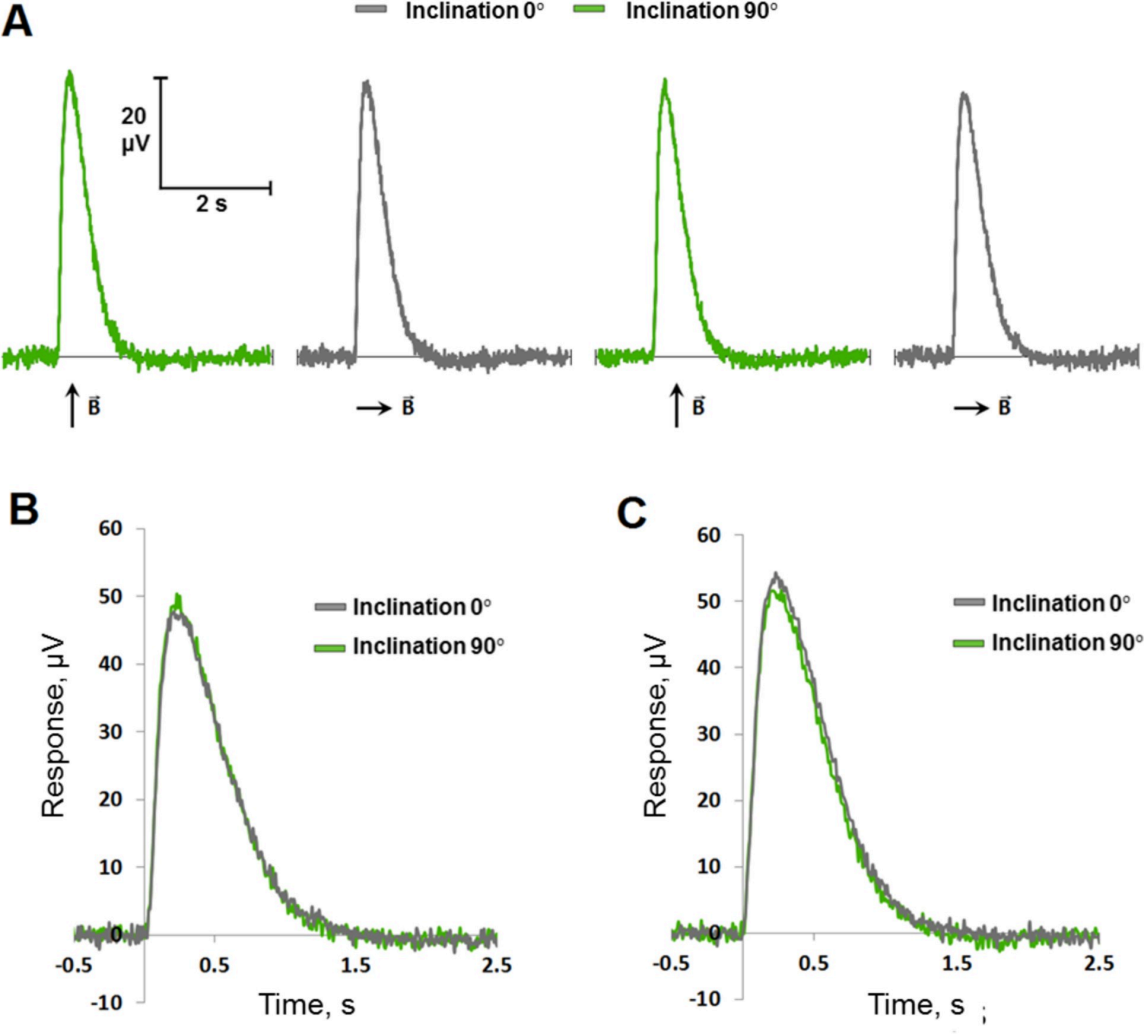
Effect of the MF inclination on photoreceptor responses from the isolated pigeon retina. (A) Series of four average photoreceptor responses to half-saturating blue flashes (10 ms, intensity 2.1×10^8^ photons/mm^2^) recorded one after another with changing of MF inclination (90°→0°→90°→0°). (B) Two average photoreceptor responses to half-saturating blue flashes (10 ms, intensity 2.1×10^8^ photons/mm^2^) at different MF inclinations. (C) Same for two average responses to half-saturating red flashes (10 ms, intensity 1.8×10^10^ photons/mm^2^).

The typical experimental protocol (Fig 3A) and representative photoreceptor responses to blue (Fig 3B) and red (Fig 3C) half-saturating flashes are shown with overlap of responses recorded under two different MF inclination values.

### Statistical analysis

To determine whether the differences in photoresponses amplitude were statistically significant, we calculated the ratio of the amplitude recorded under magnetic inclination 0° to the amplitude for inclination 90° and used the Student’s one sample t-test to compare it with 1. Shapiro-Wilk’s test was applied to confirm the normality of data distribution. If needed, the amplitudes of half-saturated responses were normalized by the maximal response amplitude, achieved for a given preparation.

The difference in the shape between two normalized responses can be quantitatively characterized by sum of the absolute values of the difference between the two responses normalized on the number of points the response records consisted of. So, to compare the kinetics of photoreceptor responses we performed the following protocol:

1. We normalized every averaged response from 2nd, 3rd and 4th sets on their own maximal amplitude to make them all have the amplitude of 1 unit. This procedure allows us to avoid the effect of amplitude changing with time on the result.
2. We calculated point-by-point differences between two normalized responses from the sets recorded either under the same inclinations of the MF or under two different inclinations for each retinal preparation and then squared these differences.
3. We summarized the squared differences for every recorded point of the response and then averaged these sums on number of terms, i.e. on number of points the response comprise of. The values of three sums were obtained: first, for the point-by-point difference between the two responses recorded under the same MF with inclination 0°, which served as a control; second, for the point-by-point difference between the first response recorded under MF with inclination 0° and the response recorded under MF with inclination 90°; third, for the difference between the second response recorded under MF with inclination 0° and the same answer recorded under MF with inclination 90°.
4. We formed three samples from described three types of averaged sums obtained for each retinal preparation (red or yellow field) and compared these samples using the one-way repeated measures ANOVA with post hoc Bonferroni correction.

All data were analyzed using Microsoft Excel 2010 (Microsoft Corp., USA), IBM SPSS Statistics 22.0 (SPSS software, IBM Corp., USA) and GraphPad Prism 8 (GraphPad software, Inc., USA). Statistical significance was set at p<0.05. N refers to number of retina preparations throughout the Results section.

The raw data for amplitude and kinetics analysis are presented both for our earlier study [15] and the current work as supporting files “S1 File” and “S2 File”, respectively.

## Results

### Decrease of the response maximum during the experimental procedure

We found that the photoreceptor responses demonstrate slight decrease in amplitude during our experimental protocol. A possible reason for this process could be slow metabolic deterioration of the retina. We tested if this decrease was statistically significant.

**Fig 4:**
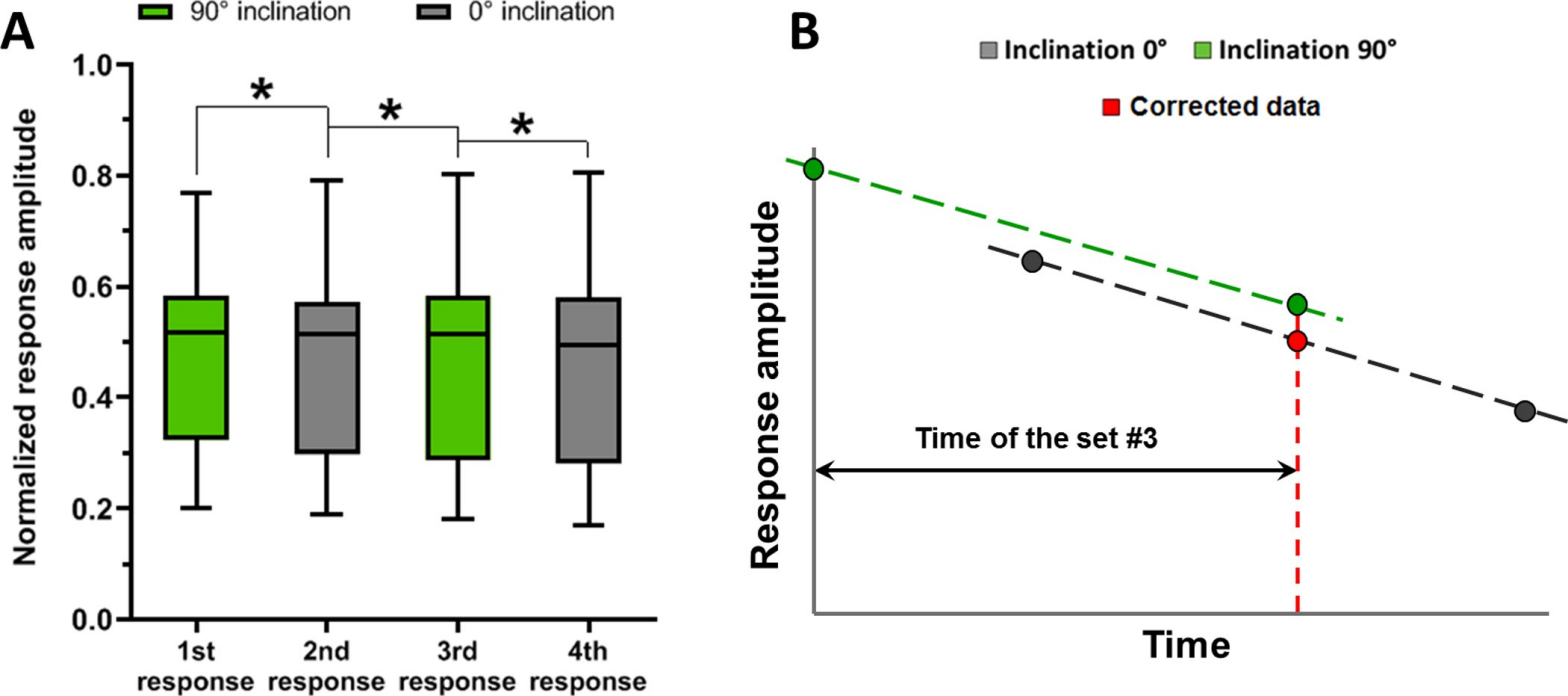
Analysis of gradual changes in the response maximum. (A) Differences in the amplitude maximum of the average responses to blue flashes recorded one after another with certain time intervals (normalized by response amplitude, achieved for a given preparation). For all retinal preparations (n = 31) responses show significant decreasing of the amplitude maximum. Data presented as medians (black horizontal lines) and quartiles (boxes and bars).*—statistically significant changes for one-way repeated measures ANOVA with *post hoc* Bonferroni correction. (B) Pair of responses recorded under the same 0° inclination of MF was used for building the linear trend in the amplitude of photoreceptor responses over time, and then such calculated trend was used for calculation of corrected response amplitude at the same time point as the amplitude for inclination 90°. The value of amplitude used for subsequent statistical analysis is marked as “corrected data”.

Statistical analysis showed that, after normalization by the maximal response amplitude, achieved for a given preparation, for the responses to blue stimulus, the 1st average (n = 31) response maximum was significantly higher than 2nd average response maximum (one-way repeated measures ANOVA with post hoc Bonferroni correction: p = 0.007). Likewise, the response maximum was significantly higher for the 2nd average response than for the 3rd (p = 0.012), and higher for the 3^rd^ average response than for the 4th (p = 0.004; see Fig 4A). We obtained a different result after analysis of the responses to red stimulus: repeated measures ANOVA showed no significant decrease in the average (n = 31, F = 0.140, p = 0.711) maximum of responses recorded at different times (see the supporting file “S2 Fig”). This result could possibly be explained by a difference in spectral sensitivity of the pigeon’s photoreceptors to our light stimuli. The sensitivity of pigeon rods to 630 nm (red) stimulus is several orders of magnitude lower than to the 470 nm (blue) stimulus [17], which means that rods contribution to the whole photoreceptor response is much lower for the responses to red flashes than to blue flashes. One could expect that rods are more sensitive to retinal preparation handling and perfusion conditions, which results in decrease of their response amplitude after several flashes. Thus, responses to blue flashes containing relatively large rod component would show a decrease in their amplitude, while responses to red flashes predominantly containing the responses of only long-wavelength sensitive cones would not show any significant amplitude decrease.

Such effect could lead to misinterpretations of our experimental data concerning photoresponse maxima, resulting in false positives. To avoid it, we performed a correction of the amplitude of the photoreceptor responses, recorded under particular inclination of MF used in experimental protocol, over time (Fig 4B). We used the previously recorded four sets of responses, where average maxima of 1st and 3rd set were used to calculate the linear trend for responses under MF with inclination 90°, average maxima of 2nd and 4th sets–for responses under the MF with inclination 0°. Still, we tested whether the slopes of these two trends are significantly different, which would correspond to non-linearity of the response amplitude changes during the time of experiment. At least in one case, for the full ERG dataset from our earlier study [15], we found a significant difference between slopes for 0° and 90° inclination linear trends (Student’s t-test for paired samples, t = 3.554, p = 0.002, see the supporting file “S3 Fig”). According to this result, we suggest that the initial part of the curve describing changes in response amplitude during time has a different slope than the remaining part. Therefore, to make our data processing method more reliable, we analyzed our data by taking into calculations only last three sets of responses (“0° – 90° – 0°” scheme). We used linear regression correction only for the amplitude of response recorded under magnetic inclination 0°. This way we found the value corresponding to the same time point as the amplitude for inclination 90°.

Despite the fact that a significant decrease in response maximum was observed only for the responses to blue flashes, the same procedure was applied to the responses to red flashes in order to standardize the data processing protocol and avoid effect that could be not significant in general, but distort data for several particular retinal preparations. Moreover, we re-analyzed the full ERG data from our earlier study according to the described protocol to ensure that the significant difference in amplitude that occurred with the change of magnetic inclination was not a false positive.

### Comparison of response maxima under different MF inclination

We analyzed the amplitudes for full ERG data from our earlier study [15] together with isolated photoreceptor potentials. The response maxima recorded under magnetic inclination 0° were corrected as described above and the ratios of the amplitude recorded under magnetic inclination 0° to the amplitude for inclination 90° were calculated. We used the Student’s one sample t-test to compare this ratio with 1, moreover, for the current study’s data we analyzed the effect of MF inclination on response maximum for red and yellow fields of the pigeon retina preparations separately.

**Fig 5:**
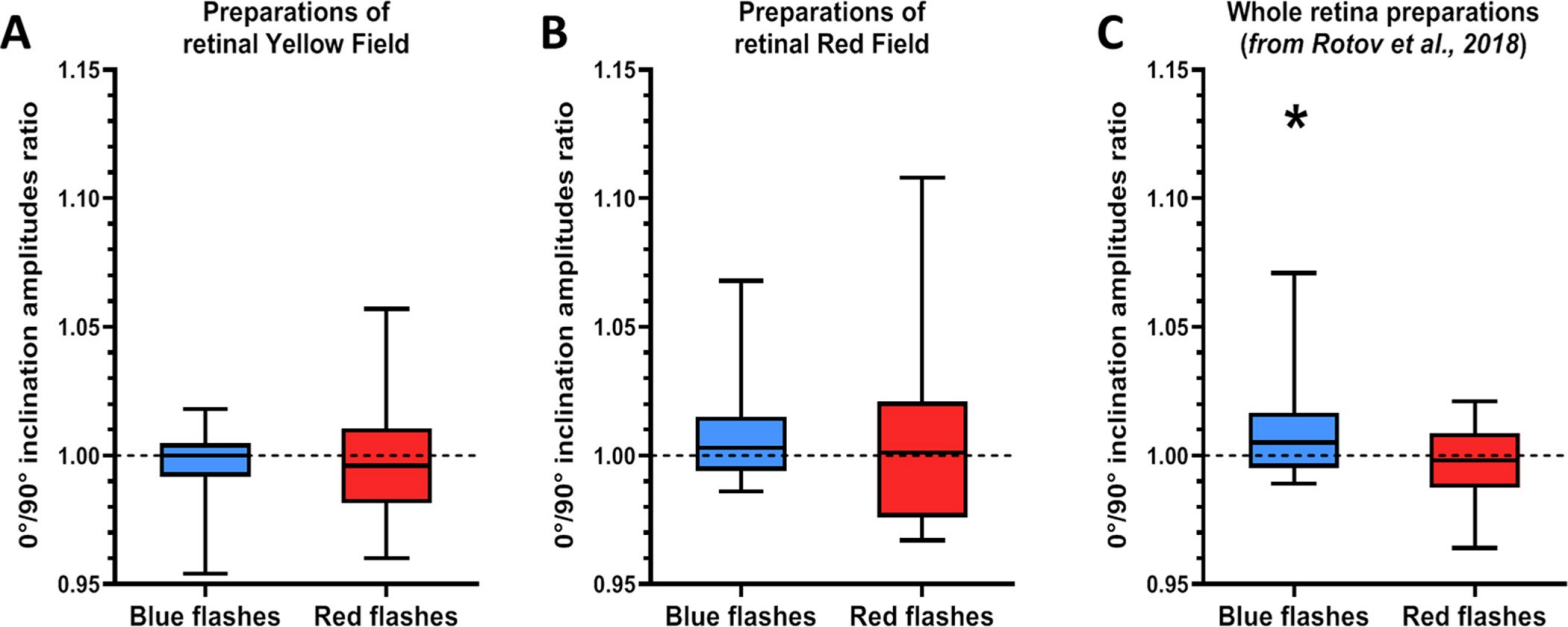
Analysis of potential effect of MF inclination change on the response maximum (amplitude). Y-axis shows the ratio of the response maximum recorded under magnetic inclination 0° to the response maximum for inclination 90°. (A) Results for yellow field preparations. n = 16 for responses both to blue and red flashes. (B) Results for red field preparations. n = 15 for responses both to blue and red flashes. For both types of retinal fields no significant changes in response maximum’s ratio were detected by Student’s one sample t-test. (C) Results for total retinal preparations from a previous study [15]. n = 20 for responses both to blue and red flashes. Ratio of amplitudes recorded under 0°/90° inclinations under blue flashes was significantly different from 1 (Student’s one sample t-test, t = 2.192, p = 0.041). For red flashes, no significant changes from 1 for response maximum’s ratio were detected. Data presented as medians (black horizontal lines) and quartiles (boxes and bars).

For the yellow field preparations the average photoreceptor response maximum amplitude to blue flashes was 50±2 μV, while to red flashes it was 52±3 μV. Half-saturating intensity varied from 4.7×10^7^ to 8.3×10^8^photons/mm^2^ for blue flashes, and from 8.3×10^9^ to 5.6×10^10^ photons/mm^2^ for red flashes. We found no significant effects of MF inclination change on the amplitude of photoreceptor responses to blue (n = 16, t = -1.045, p = 0.312) or red flashes (n = 16, t = -0.365, p = 0.720; see Fig 5A).

Average response amplitude for the preparations of the red field was 36±3 μV for blue flashes and 42±5 μV for red flashes. For blue flashes half-saturating intensity varied from 9.3×10^7^ to 2.9×10^10^ photons/mm^2^, and for red flashes–from 7×10^9^ to 7×10^10^ photons/mm^2^. There were also no significant effects of MF inclination change on the responses amplitude neither to blue (n = 15, t = 1.219, p = 0.243) nor to red flashes (n = 15, t = 0.515, p = 0.615; see Fig 5B).

The re-analysis of full ERG data from our earlier study showed generally the same pattern as the one presented in the original paper. The responses to blue flashes showed statistically significant deviation of ratio of amplitudes recorded under 0°/90° inclinations from 1, meaning that response amplitude for 0° inclination was significantly higher than amplitude for 90° (n = 20, t = 2.192, p = 0.041, see Fig 5C). Conversely, we detected no significant changes in response maximum under MF with two different inclinations for the responses to red flashes (n = 20, t = -0.812, p = 0.427).

### Comparison of response kinetics under different MF inclination

Similarly to response maximum, we analyzed the effect of MF inclination on response kinetics separately for preparations of yellow and red fields of pigeon retina. One could expect the change in sums of squares of point-by-point differences as the effect of MF inclination change on the kinetics of photoreceptor responses, so that the difference between responses with the same inclination (0°) would be significantly lower than the difference between responses with different inclinations (0° and 90°).

**Fig 6:**
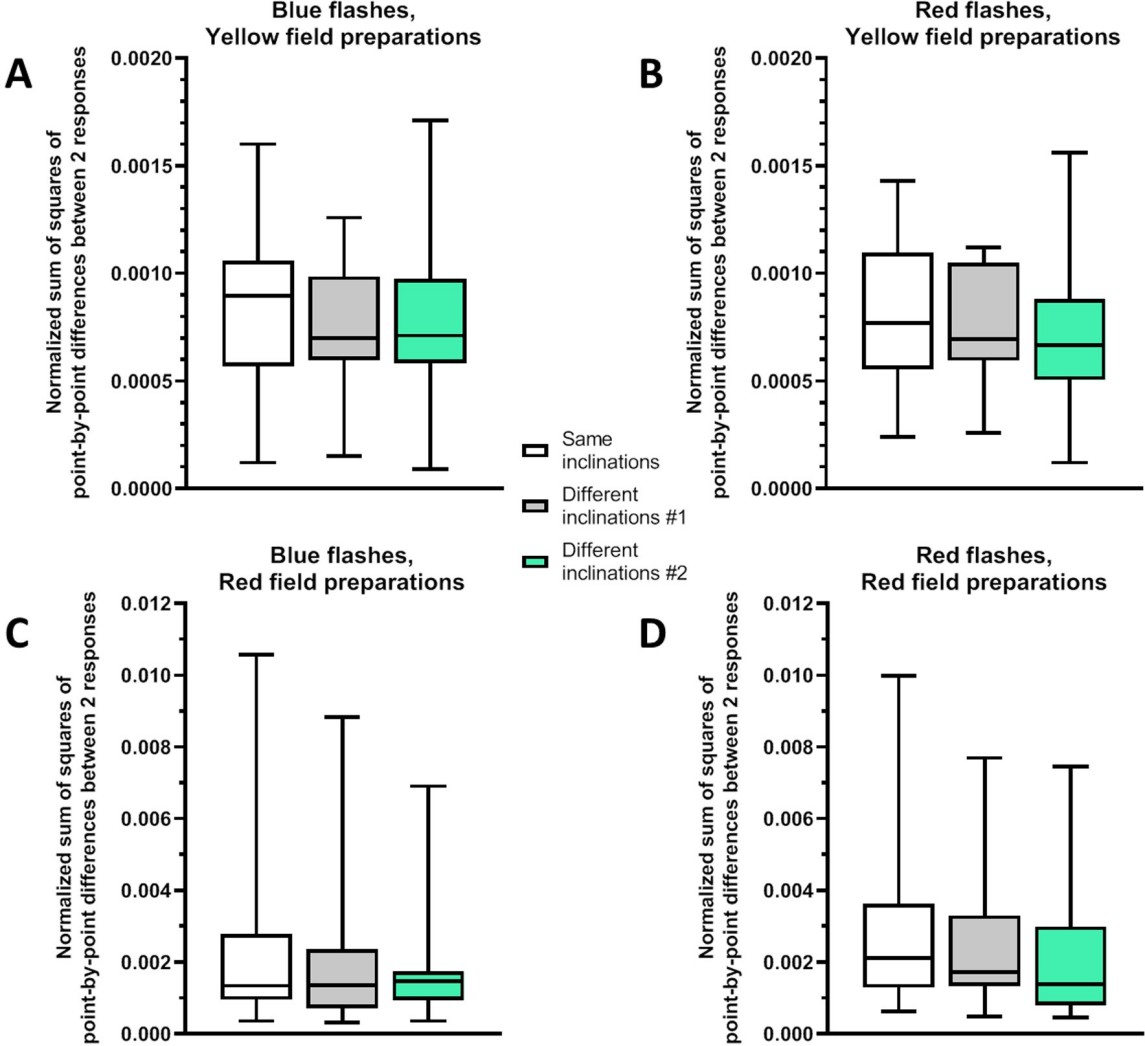
Analysis of potential effect of MF inclination change on the response kinetics. Y-axis shows average sums of point-by-point differences between normalized responses recorded under the same (0°) or under two different (0° and 90°) MF inclinations. Results presented for responses both to blue and red flashes. “Different inclination-1” and “Different inclination-2” refer to the sums of point-by-point differences between the responses recorded under the MF inclination 90° and 1st or 2nd set of responses recorded under inclination 0°, respectively. (A) Results for yellow field preparations. n = 16 for responses both to blue and red flashes. (B) Results for red field preparations. n = 15 for responses both to blue and red flashes. For both types of retinal fields no significant changes in response maximum were detected by one-way repeated measures ANOVA. Data presented as medians (black horizontal lines) and quartiles (boxes and bars).

However, we did not detect any statistically significant changes by one-way repeated measures ANOVA. Re-analysis of the data from our earlier study [15] showed the same results as the analysis performed in original paper: no changes for responses neither to blue flashes (n = 20, F = 2.378, p = 0.106) nor to red flashes (n = 20, F = 1.629, p = 0.219, see the supporting file “S4 Fig”). In the current study, for preparations of yellow retinal field we found no significant effect of MF inclination change for responses to blue (n = 16, t = 0.636, p = 0.536) or red flashes (n = 16, F = 0.664, p = 0.552; see Fig 6A and 6B). For red field preparations there was no significant effect on the kinetics of photoreceptor responses to blue (n = 15, F = 1.673, p = 0.217) or red flashes (n = 15, t = 1.573, p = 0.230; see Fig 6C and 6D), either.

## Discussion

Over the recent decades, a large body of indirect data has been accumulating that suggests the involvement of the retina in the mechanism of avian magnetic compass. However, so far no direct evidence has been presented of involvement of one or more cellular types of retina in the reception of magnetic field inclination. According to the current view, the most probable molecular candidates for the substrate of magnetoreception, cryptochromes 4 and 1b, are expressed in the photoreceptor cells of avian retina, particularly in cones [13, 25, 26]. Earlier, we performed ERG recordings on isolated retina of the pigeon without pharmacological isolation of photoreceptor potential and found a small but statistically significant influence of the MF inclination on retinal responses to blue, but not to red flashes. This result is confirmed here by the re-analysis of the data with new processing protocol. The present work was designed to test for the possibility that photoreceptor layer of isolated retina alone is able detect the MF inclination.

We analyzed the possible effect of MF inclination on the amplitude and kinetics of the response. To test that, we used half-saturating blue and red short flashes. Furthermore, the analysis was performed on two regions of pigeon retina, red and yellow fields, that differ in the proportion of long-wavelength sensitive cones. The red field is characterized by a relatively high concentration of red and orange oil droplets, i.e.by high concentration of long-wavelength sensitive cones. The role of such compartmentalization of the pigeon retina has traditionally been thought to be correlated with behavioral tasks, e.g. enhancing contrast sensitivity for objects lying against a green background for the red field, while the yellow field would provide enhanced contrast sensitivity for objects against a blue sky [27]. According to the most recent and detailed study of localization of the putative “magnetoreceptor” cryptochrome 4, it is mainly co-expressed with the long-wavelength cone visual pigment [12]. Thus one might expect a more prominent effect of MF in the red field of pigeon retina. However, we did not observe any difference in the effect of MF inclination in red and yellow field.

It should be noted that we observed no difference in spectral sensitivity between yellow and red field preparations of the retina. However, the difference in both these retinal parts' sensitivity between blue and red stimuli is consistent with absorbance spectrum of rod (but not long wavelength-sensitive cone) visual pigment (see supporting files “S5 Fig and S6 Figs”). Therefore, we can conclude that half-saturated responses observed in the present study were entirely rod-mediated. We assume that working at only rod-activating range of intensities still provided us with a possibility to detect magnetoreception which is presumably located in cone photoreceptors. According to the hypothesis proposed by Hore & Mouritsen [12], the cryptochromes (the main candidates on the role of magnetoreceptive molecule) located in cones could be activated even by low intensity stimulus and produce the electrical cell response while the visual pigment molecules stay inactive and phototransduction cascade keeps its dark state. Therefore, in the scotopic range of light intensities cones would function only as magnetoreceptors but not photoreceptors, still their response to changing MF might be detected as an addition to rod-mediated photoresponse, recorded from the retina. According to our results, no such additional component is detected in isolated photoreceptor potential.

One explanation for our results could be that magnetic compass is localized in retinal cells other than photoreceptors. Nießner and colleagues showed that CRY 1b is expressed in ganglion cells, but not in the photoreceptors of European robins *Erithacus rubecula* [28]. The recent work by Mouritsen and co-workers also identified CRY1a in garden warbler *Sylvia borin* ganglion cells and large displaced ganglion cells, i.e. not in photoreceptors [29]. It might be useful to look again at the possibility that the magnetoreception mechanism of the magnetic compass of migratory birds is localized not in photoreceptors, but in other retinal cells. Another possible explanation is that photoreceptors do participate in magnetoreception, but require some processing of compass information in other retinal layers, so that only whole retina signal can reflect the response to changing MF. Still another possibility is that only a small portion of photoreceptor cells are the detectors of MF and their signals are diluted in the pooled signal from photoreceptor layer of the entire retina, so that they are no longer detectable. This possibility should be tested by performing single cell recordings from the most promising photoreceptor cells, e.g. double cones and long-wavelength single cones [13].

## Supporting information

S1 Fig Noise spectra (per 10 kHz) recorded in the experimental room (black curve) and inside the experimental chamber (red curve) with all systems of the experimental setup running. Magnetic noise was measured using a Textronix digital oscilloscope TDS2022c equipped with a high-frequency preamplifier and a loop antenna consisting of a single turn of wire. To increase the sensitivity, we used the computer accumulation of the noise spectrum measured by the oscilloscope. Figure shows two spectra corresponding to the noise level in the laboratory (black curve) and inside the experimental chamber (red curve) when all systems of the experimental setup are running. To calculate the total field intensity in this spectral range we assume that magnetic field noise at different frequencies has a random phase and hence we use the root of the sum of the squares of the individual spectral components of the noise B_t_ = ∑_i_ √(B_i_^2^) rather than its sum to determine the total amplitude of the magnetic field. Values of the total time-dependent magnetic field intensity outside and inside the experimental chamber calculated in this way equals 28 nT and 8 nT, respectively. Grounding of the Faraday cage is done through a socket on the common ground of the institute, which is apparently rather noisy, since the screening of noise by a Faraday cage is not very effective.
(TIF)

S2 Fig Time dependence of the amplitude of responses to red flashes. Normalized amplitude maximum of the average responses to red (630 nm) flashes recorded one after another with certain time intervals are presented. For all retinal preparations (n = 31) one-way repeated measures ANOVA with post hoc Bonferroni correction did not show any statistically significant changes of response maximum. Data presented as medians (black horizontal lines) and quartiles (boxes and bars).
(TIF)

S3 Fig Comparison of the slopes of linear trends for full ERG responses to red flashes. Linear trends were built for amplitudes at 0° and 90°magnetic inclinations, respectively. Their slopes showed significant difference, which correspond to non-linearity of the whole response amplitude changes during the time of experiment (Student’s t-test for paired samples, t = 3.554, p = 0.002).Therefore, the initial part of the curve describing changes in response amplitude during time, should be excluded from the correction procedure, so we analyzed our data by taking into calculation only last three sets of responses. Data are presented as medians (black horizontal lines) and quartiles (boxes and bars).
(TIF)

S4 Fig Analysis of potential effect of MF inclination change on the full ERG kinetics. Y-axis shows average sums of point-by-point differences between normalized responses recorded under the same (0°) or under two different (0° and 90°) MF inclinations. “Different inclination-1” and “Different inclination-2” refer to the sums of point-by-point differences between the responses recorded under the MF inclination 90° and 1st or 2nd set of responses recorded under inclination 0°, respectively. (A) Results for responses to blue flashes. (B) Results for responses to red flashes. For both stimuli no significant changes in response maximum were detected by one-way repeated measures ANOVA (n = 20). Data presented as medians (black horizontal lines) and quartiles (boxes and bars).
(TIF)

S5 Fig Dependencies of normalized response maximum on light intensities for preparations of yellow and red field of the pigeon retina. Individual points indicate values for individual preparations. Student’s t-test for two samples did not show any statistically significant difference in sensitivity between retinal red (n = 15) and yellow (n = 17) field preparations either for red or blue stimuli. Thus, the difference in proportion of long wavelength-sensitive cones between these fields does not influence their sensitivities to blue and red stimuli with intensities used in our experimental protocol.
(TIF)

S6 Fig Superimposition of absorbance spectra of visual pigments (green solid line represents visual pigment of pigeon rod, red solid line represents visual pigment of pigeon red-sensitive cones) and emission spectra of two LED (blue dashed line–blue LED, red dashed line–red LED). Sensitivity of visual pigment to the particular LED can be estimated from overlap areas between respective absorbance and emission spectra. The ratio of sensitivity between blue and red LEDs is approximately 1:1 for red-sensitive cones and 100:1 for rod visual pigment. Supporting file “S5 Fig” indicates that approximately half-saturating intensities used in present study correspond to ratio of sensitivity between blue and red flashes about 100:1. It means that half-saturated responses observed in the present study are entirely rod-mediated.
(TIF)

S1 File Raw data for the full ERG responses (for MF magnitude 50 μT). This table contains all raw data on amplitudes and kinetics for the results originally described in our earlier study [15]. Amplitudes presented as averaged from each of four sets (with corresponding times), also as corrected values for amplitudes at 0° MF inclination and maximal response amplitude, achieved for a given preparation. The kinetics data presented as averaged sums of point-by-point differences between the normalized responses recorded under the MF inclination 0° and 90°.
(XLSX)

S2 File Raw data for the isolated photoreceptor potential. This table contains all raw data on amplitudes and kinetics for the results described in current study. Amplitudes presented as averaged from each of four sets (with corresponding times), also as corrected values for amplitudes at 0° MF inclination and maximal response amplitude, achieved for a given preparation. The kinetics data presented as averaged sums of point-by-point differences between the normalized responses recorded under the MF inclination 0° and 90°.
(XLSX)
