# Supplementary figures and images for: Searching for magnetic compass mechanism in pigeon retinal photoreceptors
Source: PLoS One. 2020 Mar 5;15(3):e0229142. doi: 10.1371/journal.pone.0229142 (PMC7058337; doi:10.1371/journal.pone.0229142)

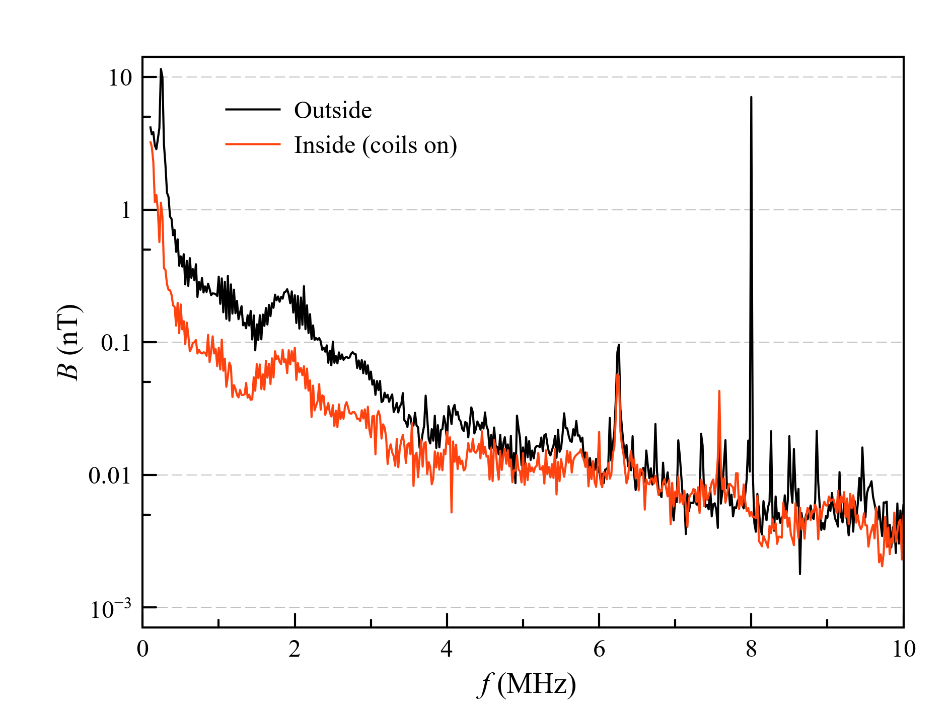

Supplement: S1 Fig — Magnetic noise was measured using a Textronix digital oscilloscope TDS2022c equipped with a high-frequency preamplifier and a loop antenna consisting of a single turn of wire. To increase the sensitivity, we used the computer accumulation of the noise spectrum measured by the oscilloscope. Figure shows two spectra corresponding to the noise level in the laboratory (black curve) and inside the experimental chamber (red curve) when all systems of the experimental setup are running. To calculate the total field intensity in this spectral range we assume that magnetic field noise at different frequencies has a random phase and hence we use the root of the sum of the squares of the individual spectral components of the noise Bt = ∑i √(Bi2) rather than its sum to determine the total amplitude of the magnetic field. Values of the total time-dependent magnetic field intensity outside and inside the experimental chamber calculated in this way equals 28 nT and 8 nT, respectively. Grounding of the Faraday cage is done through a socket on the common ground of the institute, which is apparently rather noisy, since the screening of noise by a Faraday cage is not very effective. (TIF) [file pone.0229142.s001.tif]

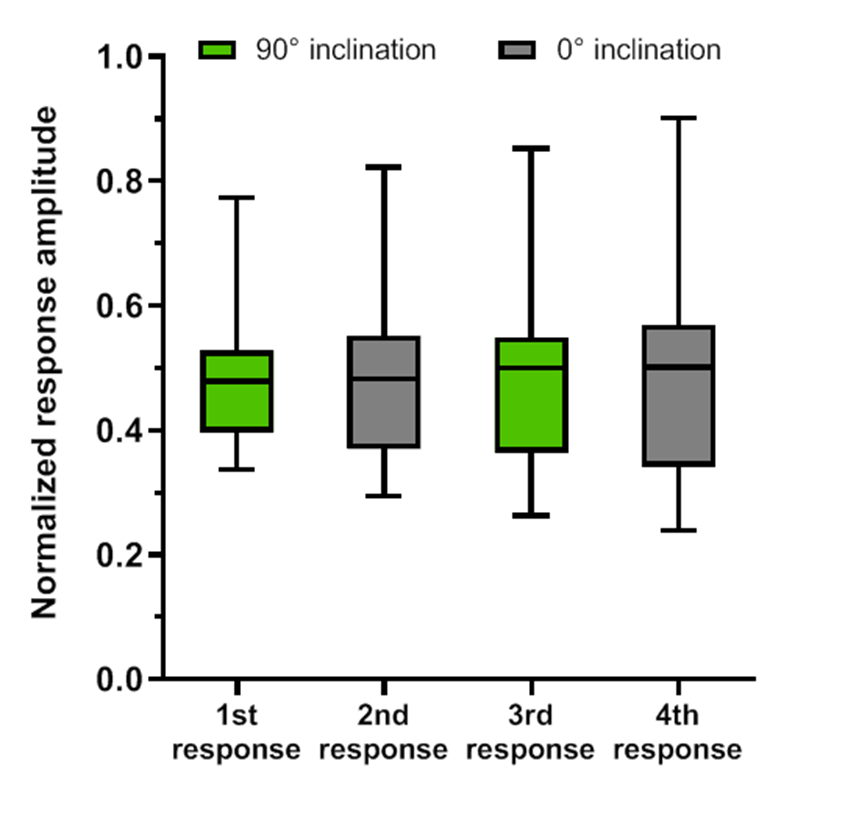

Supplement: S2 Fig — Normalized amplitude maximum of the average responses to red (630 nm) flashes recorded one after another with certain time intervals are presented. For all retinal preparations (n = 31) one-way repeated measures ANOVA with post hoc Bonferroni correction did not show any statistically significant changes of response maximum. Data presented as medians (black horizontal lines) and quartiles (boxes and bars). (TIF) [file pone.0229142.s002.tif]

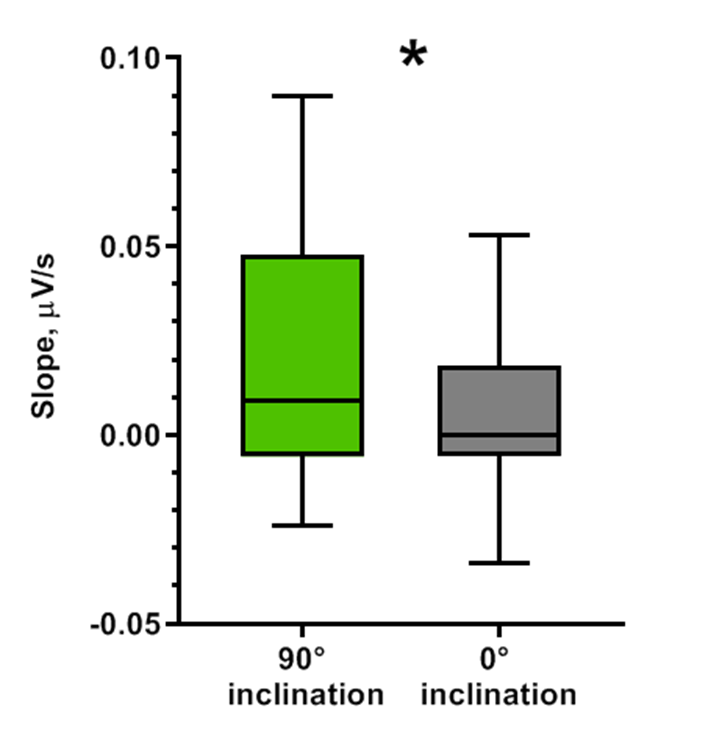

Supplement: S3 Fig — Linear trends were built for amplitudes at 0° and 90°magnetic inclinations, respectively. Their slopes showed significant difference, which correspond to non-linearity of the whole response amplitude changes during the time of experiment (Student’s t-test for paired samples, t = 3.554, p = 0.002).Therefore, the initial part of the curve describing changes in response amplitude during time, should be excluded from the correction procedure, so we analyzed our data by taking into calculation only last three sets of responses. Data are presented as medians (black horizontal lines) and quartiles (boxes and bars). (TIF) [file pone.0229142.s003.tif]

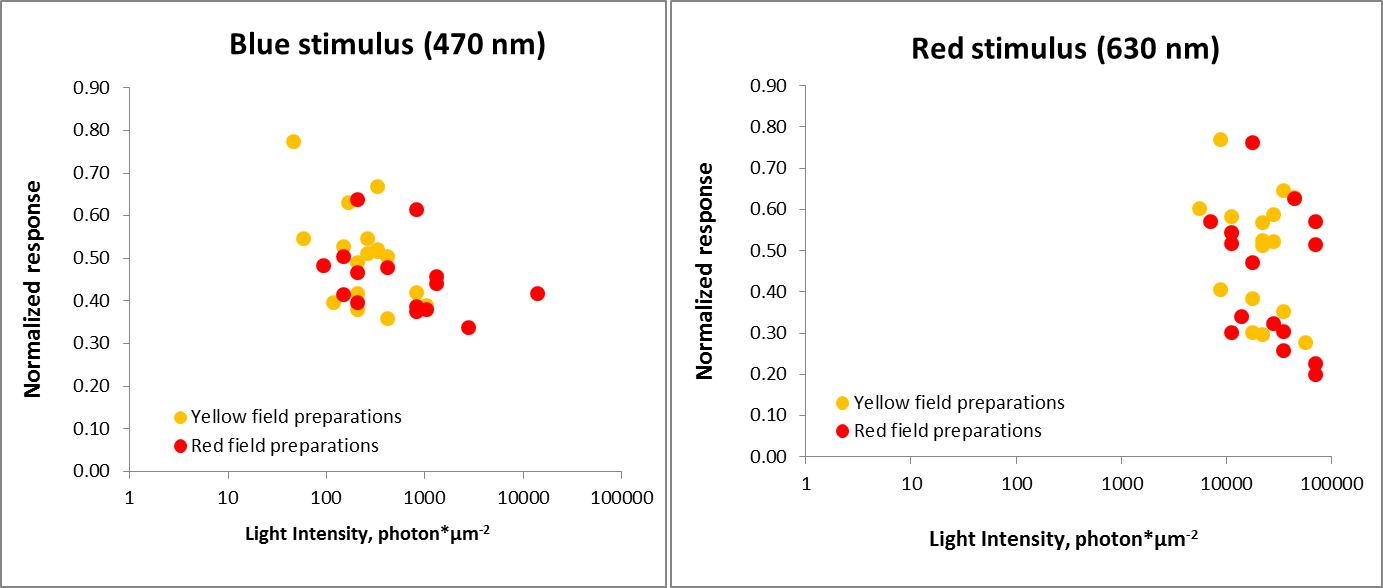

Supplement: S5 Fig — Individual points indicate values for individual preparations. Student’s t-test for two samples did not show any statistically significant difference in sensitivity between retinal red (n = 15) and yellow (n = 17) field preparations either for red or blue stimuli. Thus, the difference in proportion of long wavelength-sensitive cones between these fields does not influence their sensitivities to blue and red stimuli with intensities used in our experimental protocol. (TIF) [file pone.0229142.s005.tif]

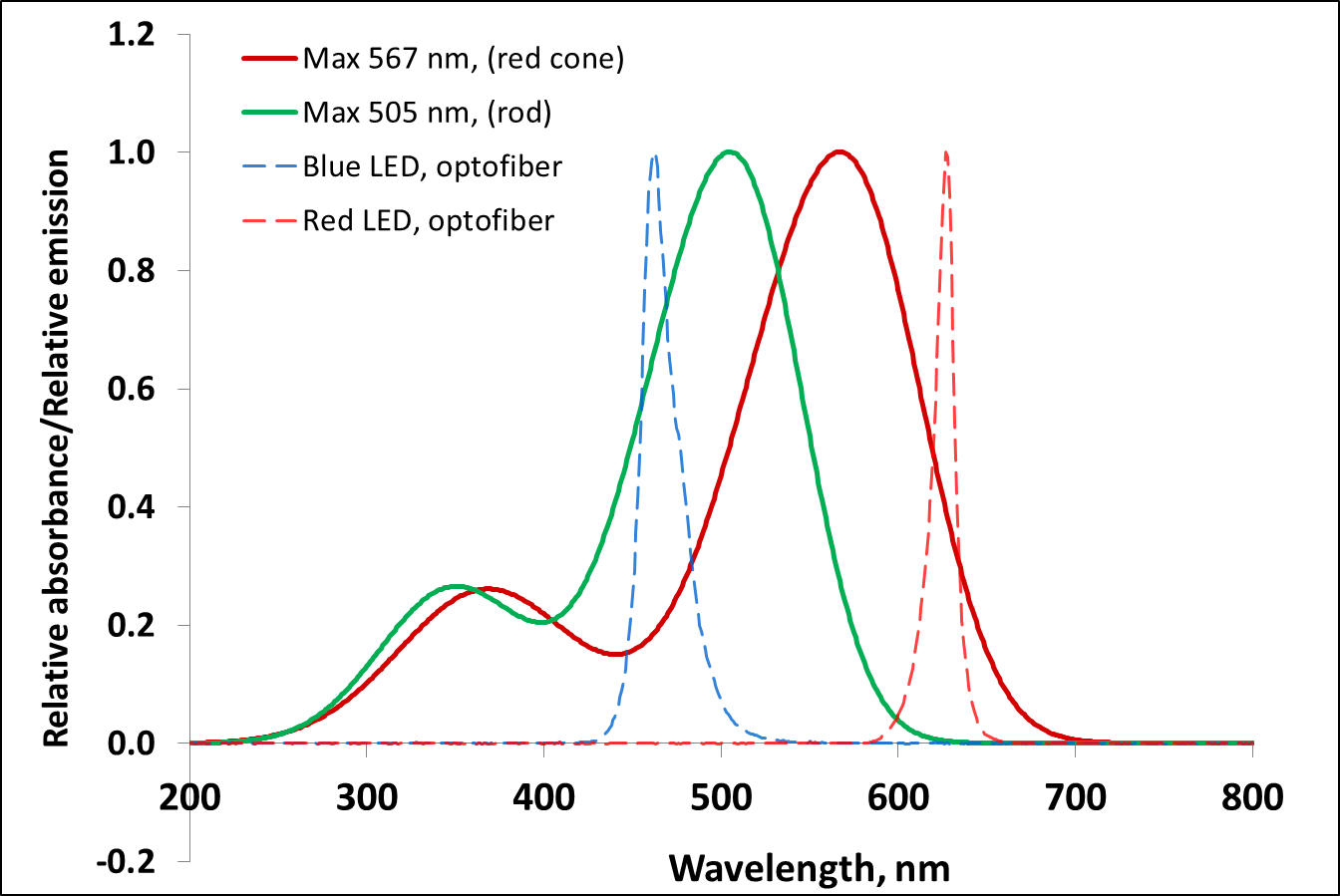

Supplement: S6 Fig — Sensitivity of visual pigment to the particular LED can be estimated from overlap areas between respective absorbance and emission spectra. The ratio of sensitivity between blue and red LEDs is approximately 1:1 for red-sensitive cones and 100:1 for rod visual pigment. Supporting file “S5 Fig” indicates that approximately half-saturating intensities used in present study correspond to ratio of sensitivity between blue and red flashes about 100:1. It means that half-saturated responses observed in the present study are entirely rod-mediated. (TIF) [file pone.0229142.s006.tif]
